# P101 From chronic immune dysregulation to acute hyperinflammation: a case of lymphocytic interstitial pneumonia and haemophagocytic lymphohistiocytosis in primary Sjögren’s disease

**DOI:** 10.1093/rap/rkag113.131

**Published:** 2026-11-03

**Authors:** Urwah Ahmed, Marvi Iftikhar, Kriti Sharma, Clare MacEwen, Katerina Panopoulou, Alex Mentzer, Udaya Udayaraj, Yen Lim, Shirish Dubey

**Affiliations:** Oxford University Hospitals, Oxford, United Kingdom; Oxford University Hospitals, Oxford, United Kingdom; Oxford University Hospitals, Oxford, United Kingdom; Oxford University Hospitals, Oxford, United Kingdom; Oxford University Hospitals, Oxford, United Kingdom; Oxford University Hospitals, Oxford, United Kingdom; Oxford University Hospitals, Oxford, United Kingdom; Oxford University Hospitals, Oxford, United Kingdom; Oxford University Hospitals, Oxford, United Kingdom

## Abstract

**Introduction:**

Primary Sjögren’s disease (pSjD) is characterised by multi-organ chronic immune dysregulation, most often affecting the exocrine glands. Lymphocytic interstitial pneumonia (LIP) is an uncommon form of interstitial lung disease (ILD), classically associated with pSjD, while haemophagocytic lymphohistiocytosis (HLH) represents a rare but potentially fatal state of uncontrolled immune hyperactivation. Although both may occur in pSjD, their presentation in the same patient is rare. We present a case of pSjD complicated by LIP and subsequent likely infection-triggered HLH, highlighting the spectrum of immune dysregulation that can occur in this disease and the diagnostic challenges posed by acute clinical deterioration.

**Case description:**

A 34-year-old female with chronic kidney disease (Stage 5) secondary to autosomal dominant polycystic kidney disease (ADPKD) and unrelated renal tubular acidosis (RTA) presented with sicca symptoms and Raynaud’s phenomenon. Serology demonstrated rheumatoid factor positivity (188 IU/mL), ANA >1:640, hypergammaglobulinaemia (IgG 38 g/L), anti-Ro and anti-La positivity, and dsDNA positivity (100 IU/mL) by ELISA, leading to a diagnosis of pSjD.

During assessment for peritoneal dialysis (PD) and renal transplantation, a CT abdomen incidentally identified pulmonary abnormalities. CT thorax demonstrated pulmonary cysts, peribronchial consolidation and diffuse ground-glass change. Multidisciplinary respiratory review concluded that the appearances were most consistent with LIP, although chronic infection required exclusion (bronchoscopy detected low levels of Pneumocystis jiroveci (PJP) DNA and Streptococcus viridans). Pulmonary function testing showed preserved spirometry (FEV1 80% predicted, FVC 83.6% predicted with reduced gas transfer (TLCO 74.2% predicted)). Initiation of mycophenolate mofetil (MMF) was deferred because of planned travel and PD catheter insertion.

Two weeks following PD catheter placement, she was admitted with fever, tachycardia, abdominal pain and vomiting. PD peritonitis was excluded following fluid analysis. Previous bronchoscopy results were not thought to be relevant, and only positive microbiology was Escherichia coli bacteriuria. Despite broad-spectrum antibiotics, she deteriorated with persistent inflammation (CRP >200 mg/L), pancytopenia and worsening liver function tests. HLH was suspected (HScore 212, supported by ferritin 32,000 ng/mL, triglycerides >7 mmol/L); bone marrow biopsy demonstrated haemophagocytes. Genetic testing for HLH (sent due to younger age and known ADPKD) was negative. She was discussed within the local HLH MDT and treated with intravenous methylprednisolone 1mg/kg, intravenous anakinra (equivalent to 8mg/kg for normal renal function) and broad-spectrum antimicrobials/ antifungals/PJP prophylaxis; with rapid clinical and biochemical improvement. She was discharged on tapering prednisolone and subcutaneous anakinra. Subsequently, recurrence of chest and joint symptoms prompted initiation of MMF.

**Discussion:**

Although pSjD is often perceived as a relatively benign disease characterised predominantly by sicca symptoms, it is a systemic autoimmune disorder capable of causing severe immune-mediated complications. This case was complicated by RTA, LIP and HLH; illustrating the breadth of immune dysregulation that can occur, manifesting as both chronic lymphoproliferative organ disease and acute cytokine driven life-threatening hyperinflammation.

LIP is an uncommon form of interstitial lung disease characterised by diffuse lymphocytic infiltration of the pulmonary interstitium. Although most strongly associated with pSjD, it may also occur in the context of chronic infection, other autoimmune diseases and drug exposure. In this case, the radiological appearances, together with the established diagnosis of pSjD and accompanying serological profile, were most consistent with LIP. Nevertheless, bronchoscopy to exclude chronic infection proved valuable, particularly when the patient subsequently developed acute systemic deterioration.

Although recent BSR guidelines have contributed to increasing recognition of HLH in adult rheumatology practice, this diagnosis remains challenging and is frequently delayed. This is in part due to its clinical features overlapping with sepsis, particularly when plausible infective precipitants are present, as in this case following recent peritoneal dialysis catheter insertion and positive microbiological findings. CXCL9 is a specific biomarker for interferon-gamma driven hyperinflammation and can help differentiate HLH from sepsis but is currently only available in highly specialised and research settings. Whilst the HLH episode was considered most likely to be infection-triggered, it is perhaps better considered as a “second-hit” phenomenon occurring on a background of pre-existing immune dysregulation. pSjD is characterised by persistent immune activation and loss of immunological tolerance, creating an environment in which additional physiological insults may precipitate an exaggerated and self-sustaining inflammatory response. Importantly, irrespective of the precipitating cause, successful outcomes depend upon early recognition and the prompt initiation of immunomodulatory therapy alongside treating potential triggers.

**Key learning points:**

1. pSjD is a systemic autoimmune disease with manifestations extending beyond exocrine gland dysfunction. Whilst it often has a benign course and patients frequently do not receive systemic treatments; this case illustrates the importance of ensuring that patients with pSjD are provided enough care and attention to appropriately diagnose and treat potentially life-threatening complications.

2. LIP should be considered in patients with pSjD who develop cystic lung disease; however, alternative aetiologies, particularly emphysema and chronic infection, must be excluded.

3. HLH is a rare but life-threatening complication of primary Sjögren’s disease, described predominantly in case reports and small case series, underscoring the need for further research. Its diagnosis is frequently delayed because its clinical features closely resemble sepsis, particularly when potential infective triggers are present. Maintaining a high index of suspicion, undertaking early diagnostic assessment (including ferritin, triglycerides, H-score calculation and, where appropriate, bone marrow examination), and improving awareness among both healthcare professionals and patients are essential to facilitate earlier diagnosis and reduce morbidity and mortality.

4. Whilst infection is considered to be the commonest precipitant in connective tissue disease- associated HLH, the presence of a potential infective trigger should not preclude consideration of HLH or delay the initiation of immunomodulatory therapy when clinical suspicion is high; using a multidisciplinary team approach. The increasing use of interleukin-1 blockade with anakinra, which has a favourable safety profile even when infection has not been fully excluded, offers an important therapeutic option while investigations to identify the underlying trigger continue in parallel. Early use of anakinra may avoid prolonged high dose corticosteroids, which increase secondary infection risk, a significant contributor to morbidity and mortality in HLH.

